# Mechanisms of Synaptic Vesicle Exo- and Endocytosis

**DOI:** 10.3390/biomedicines10071593

**Published:** 2022-07-04

**Authors:** Sumiko Mochida

**Affiliations:** Department of Physiology, Tokyo Medical University, Tokyo 160-8402, Japan; mochida@tokyo-med.ac.jp

**Keywords:** action potential, active zone, Ca^2+^ channels, Ca^2+^ sensor proteins, exocytosis, endocytosis, presynaptic short-term plasticity, synaptic vesicle

## Abstract

Within 1 millisecond of action potential arrival at presynaptic terminals voltage–gated Ca^2+^ channels open. The Ca^2+^ channels are linked to synaptic vesicles which are tethered by active zone proteins. Ca^2+^ entrance into the active zone triggers: (1) the fusion of the vesicle and exocytosis, (2) the replenishment of the active zone with vesicles for incoming exocytosis, and (3) various types of endocytosis for vesicle reuse, dependent on the pattern of firing. These time-dependent vesicle dynamics are controlled by presynaptic Ca^2+^ sensor proteins, regulating active zone scaffold proteins, fusion machinery proteins, motor proteins, endocytic proteins, several enzymes, and even Ca^2+^ channels, following the decay of Ca^2+^ concentration after the action potential. Here, I summarize the Ca^2+^-dependent protein controls of synchronous and asynchronous vesicle release, rapid replenishment of the active zone, endocytosis, and short-term plasticity within 100 msec after the action potential. Furthermore, I discuss the contribution of active zone proteins to presynaptic plasticity and to homeostatic readjustment during and after intense activity, in addition to activity-dependent endocytosis.

## 1. Introduction

Synaptic transmission is mediated by the exocytosis of neurotransmitters filled within synaptic vesicles (SVs) towards postsynaptic receptors [1,2]. This process is initiated by a presynaptic action potential (AP) that opens voltage-gated Ca^2+^ (Ca_V_) channels composed of a neurotransmitter release site termed the active zone (AZ) [1,2]. Ca^2+^ sensor proteins expressed on the SV membrane and the SV fusion machinery of the soluble *N*-ethylmaleimide-sensitive-factor attachment receptor proteins (SNAREs) complex co-operationally mediates the fusion of neurotransmitter-containing SVs with the presynaptic plasma membrane [1,2]. The AZ is a highly organized structure composed of protein complex [1,2,3] that regulates the rapid replenishment of neurotransmitter release sites with SVs after exocytosis, for sustainable synaptic transmission [4]. This process is followed by various types of endocytosis, dependent on the pattern of AP firing, to retrieve the plasma membrane or the fused synaptic vesicle for vesicle reuse [5,6]. These time-dependent vesicle dynamics are controlled by presynaptic Ca^2+^ sensor proteins, regulating AZ scaffold proteins, fusion machinery proteins, motor proteins, endocytic proteins, several enzymes, and even Ca^2+^ channels, following the decay of Ca^2+^ concentration after AP(s), which contributes to presynaptic short-term plasticity [7].

This review introduces, at first, the current models for AZ protein assembly [8,9,10], and then the recent findings in exocytosis [11,12]. I also discuss the temporal regulation of defined states of SV within 100 msec of AP following the evoked exocytosis [13]. Replenishment of the AZ with release-ready SVs is Ca^2+^ dynamics-dependent, and involves multiple protein reactions, including calmodulin binding [14] and phosphorylation [15,16]. These protein reactions modulate exocytosis, and induce presynaptic short-term plasticity [9,12,13,14] and homeostatic synaptic plasticity [17,18,19]. I finally discuss new aspects of endocytosis [5,6,7].

## 2. Structure and Function of Presynaptic Neurotransmitter Release Sites

**Figure 1:**
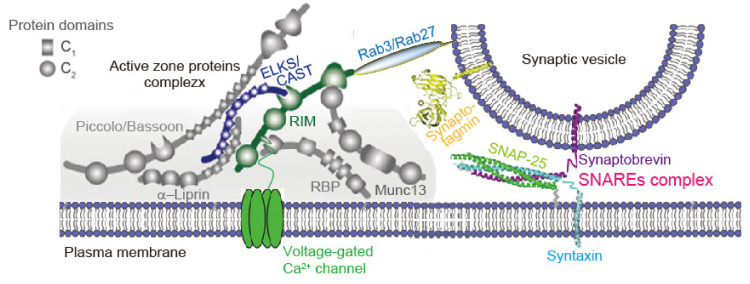
Presynaptic active zone assembly model and synaptic vesicle fusion machinery proteins. Diagram represents active zone (AZ) proteins’ complex in a liquid droplet (gray) and a docked synaptic vesicle (SV). A recent in vitro study indicates that SVs coat the surface of condensed liquid droplets [37]. In the droplet, the AZ is a highly organized structure that recruits voltage-gated Ca^2+^ channel and docks SV. Reproduced with permission from ref. [3]. Copyright 2016, Wang et al. Priming factors promote proper assembly consisting of the pre-fusion state of the fusion machinery protein complex and SNAREs/synaptotagmin/complexin (not shown) [38]. The tight spatial organization ensures fast exocytosis upon Ca^2+^ entry, and it provides molecular machinery to set and regulate synaptic strength, presynaptic short-term plasticity, and homeostatic synaptic plasticity. For detailed order of events of vesicle tethering, docking and fusion, please refer to reviews [11,39].

Within a synapse, neurotransmitter release is restricted to specialized presynaptic structures called AZs [4,8]. The AZ is a highly organized structure composed of sophisticated protein machinery (Figure 1) [1,2,3]. The AZ serves as a platform for SV exocytosis. Synaptotagmins, Ca^2+^ sensor proteins expressed on the SV membrane, mediate SV exocytosis cooperating with the SNAREs complexes, fusion machinery proteins, near Ca_V_ channels (Figure 1). The tight spatial organization enables to induce the synchronous SV fusion upon Ca^2+^ entry, and sets the synaptic strength [20].

### 2.1. AZ Structure

AZ proteins, including Munc13, RIM, RIM-BP, CAST/ELKS, Bassoon, Piccolo, and Liprin-α [21,22,23,24,25,26,27,28], are all relatively large proteins, and form a large macromolecular complex interacting with each other via significant domain structures (Figure 1) [1]. RIM (Rab3-interacting molecules) [28] and RIM-BP (RIM-binding protein) [29] are essential for the inclusion of Ca_V_ channels [30,31]. A molecular complex consisting of RIM and the C-terminal tails of the Ca_V_ channels, which determines the recruitment of Ca_V_2 channels to the AZ, includes RIM-BP and CAST (cytomatrix at the active zone-associated structural protein) [24]/ELKS [32]. In contrast, interaction of RIM, RIM-BP, and CAST/ELKS may be essential for the constitution of the AZ structure: RIM and ELKS double deletion induces the loss of Munc13-1, Bassoon, Piccolo, RIM-BP2, and the Ca_V_2 channels [3]. CAST controls Ca_V_ channel density [33], AZ size [34,35], and SV docking [15]. The disruption of CAST interaction with RIM impairs synaptic transmission [25]. Among four Liprin-α, Liprin-3α, which is strongly expressed in the brain, has recently been reported to control the co-recruitment of RIM and Mun 13 via protein kinase C (PKC)-mediated phosphorylation [36].

### 2.2. AZ Proteins Control SV States

The AZ proteins play a central role in neurotransmitter release by localizing Ca_V_ channels in the release sites as discussed above, and also in setting the defined states of SVs termed as tethering, docking, priming, and fusion [1]. Bassoon and Piccolo mediate SV tethering and regulate SV reloading [40,41,42]. RIM seems to be a key protein for SV dynamics [43,44]. RIM mediates the linkage of docked SV via Rab3/Rab27, synaptic vesicle proteins and members of the family of low-molecular weight guanosine triphosphatases (GTPases) [39,45,46], and Ca_V_ channels in the neurotransmitter release site [47] (Figure 1). Removing RIM removes SV docking and slows down the exocytosis speed [3]. RIM also mediates SV priming [48,49], leading to a SV that can rapidly fuse upon Ca^2+^ stimulation [39]. SV priming is also mediated by ELKS, RIM-BP, and Munc13 [48,49,50,51]. In addition, the involvement of Mover, another AZ protein, in the super-priming of SVs, was recently reported [52]. Munc13 and Munc18 interact with the fusion machinery and regulate exocytosis [53,54,55,56,57] (see Section 3.3). For more complete reviews on molecular mechanisms, please refer to recent articles [4,8].

### 2.3. AZ Protein Complex Formation

To explore AZ assembly models AZ protein interactions have long been examined (Figure 1) [1,3]. Except for RIM and RIM-BP, which are required for Ca_V_ channel clustering [30,58], however, deletion of single-AZ proteins causes only mild effects on the AZ structure, suggesting that individual protein interactions unlikely have a major role in the AZ assembly [8]. A recent in vitro study has proposed a new model whereby AZ assembly relies on liquid–liquid phase separation principles [59]. RIM, RIM-BP, and Ca_V_ channels form dense clusters on the supported lipid membrane bilayers via phase separation. Liprin-3α, which co-recruits RIM and Mun 13 via PKC-mediated phosphorylation, also undergoes phase separation in transfected HEK cells [36]. The new model suggests that multiple low-affinity interactions very likely drive AZ formation [8], as the suggested models of droplet-like condensate formation with protein interactions in clustering of SVs [60,61] and postsynaptic density [62]. Liquid–liquid phase separation or low-affinity interaction can contribute to membrane anchoring [37,60].

### 2.4. A Possible Model for SV Pools Formation

In presynaptic terminals or boutons four functional pools of SVs are organized: the readily releasable pool (RRP), the recycling pool, and the reserve and resting pools [5,63]. To maintain sustainable neurotransmitter release, the RRP has to be replenished constantly with SVs. During prolonged neuronal activity the replenishment is achieved by either the rapid reuse of fused vesicles, or the recruitment of new SVs from the reserve pool [5]. All SVs which participate in activity-induced neurotransmitter release comprise the recycling pool [63].

The AZ proteins RIM, RIM-BP, and ELKS form condensed liquid droplets via phase separation. SVs from rat brains and small unilamellar vesicles (SUVs) coat the surfaces of the condensed liquid droplets [37]. The SUV-coated RIM/RIM-BP condensates enable to cluster Ca_V_ channels anchored on membranes. Strikingly, synapsin/SUV condensates wrap SUV-coated RIM/RIM-BP condensates. The formation of two distinct SUV pools is likely the reserve and tethered SV pools in presynaptic boutons [37]. These studies provide a possible model for SV pools formation reconstituting a presynaptic bouton-like structure that mimics the RRP with the SV-tethered AZ and the connected reserved pool with the synapsin-clustered SV condensates.

### 2.5. AZ Assembly Stability

Bassoon and Piccolo maintain synapse integrity by regulating protein ubiquitination and degradation. The aberrant degradation of multiple presynaptic proteins is induced by loss of Bassoon and Piccolo. Loss of Bassoon and Piccolo also lead to synapse degeneration, mediated in part by the E3 ubiquitin ligase Siah1 that is an interacting partner of Bassoon and Piccolo [64]. In boutons lacking Bassoon and Piccolo the destruction of SVs is associated with presynaptic autophagy, a process dependent on poly-ubiquitination. The gain or loss of function of Bassoon alone suppressed or enhanced presynaptic autophagy, respectively, indicating a fundamental role for Bassoon in the local regulation of presynaptic autophagy [41]. Bassoon and Piccolo are critical regulators for stabilizing the AZ assembly by inhibiting degradation.

## 3. Synaptic Vesicle Exocytosis

SV fusion is a series of events, and its flow is controlled by a number of protein–protein interactions: Fusogenic SNAREs, the Ca^2+^-sensor synaptotagmin, the activator/regulator complexin, the assembly factors Munc18 and Munc13, and the disassembly factors NSF and SNAP have been identified as key factors of the core synaptic fusion machinery [11].

### 3.1. SV Fusion Complex—A Model where Ca^2+^ Releases Inhibition of SV Fusion

Synaptotagmins expressed on the SV membrane cooperating with the SNAREs complex has been implicated to mediate the fusion of neurotransmitter-containing SVs with the presynaptic plasma membrane [1,2]. Recent structural and functional studies suggest that AP-evoked sub-millisecond SV fusion occurs with the release of inhibition by Ca^2+^ binding to synaptotagmin [38,65]. Complexin, a cytoplasmic protein that is crucial for the regulation of neurotransmitter release [66,67], contributes to the inhibition process. The structure of SNAREs, synaptotagmin-1, and complexin-1 complex clarifies two interfaces: the primary interface between synaptotagmin-1 and SNAREs, and a tripartite interface between SNAREs, complexin-1, and synaptotagmin-1. These two interfaces of the complex suggest the cooperation of all three components in the evoked SV fusion [11]. The tripartite complex of SNAREs/complexin/synaptitagmin-1 is formed in the absence of Ca^2+^, suggesting a prefusion complex inhibiting SV fusion [11].

Synaptotagmins contain cytoplasmic C2A and C2B domains [68,69]. The structure of SNAREs, complexin-1, and synaptotagmin-1 complex clarifies a tripartite interface between one synaptotagmin-1 C2B domain, SNAREs and complexin-1 [65]. This tripartite interface formation is promoted by complexin-1 binding to SNAREs. The second synaptotagmin-1 C2B domain simultaneously interact with the other side of the SNAREs via a pairwise interface. Synaptotagmin-1 C2B residues is responsible for the Ca^2+^-triggered synchronous neurotransmitter release and the suppression of spontaneous release. Synaptotagmin-1 C2B residues are involved in both the tripartite interface of SNAREs, complexin-1 and synaptotagmin-1 and the primary interfaces of SNAREs and synaptotagmin-1 [11,65,70].

Synaptotagmin-2, at the calyx of the Held synapse and in some GABAergic neurons, regulates neurotransmission redundantly with synaptotagmin-1 [71,72]. Synaptic transmission depends on synaptotagmin-1 in the early postnatal calyx of the Held synapses, but later switches to synaptotagmin-2 [72], suggesting dynamic changes in synaptotagmin content at the synapses during development. In the hippocampal neurons, synaptotagmin-7 acts redundantly with synaptotagmin-1 in the maintenance of the RRP of SVs [73]. The loss of synaptotagmin-1 function in immature neurons can compensate with the expression of synaptotagmin-7 [12]. The structural study of the tripartite complex of SNAREs/complexin/synaptitagmin-1 has not yet been applied for synaptotagmin-2 or -7 in the fusion machinery.

### 3.2. Asynchronous SV Fusion

Ca^2+^-triggered evoked SV fusion causes synchronous neurotransmitter release, which occurs within tens of microseconds of a stimulus and completes within several milliseconds [74], transmitting a fast and reliable signal. The evoked SV fusion also causes asynchronous neurotransmitter release, which sets in more slowly and can persist for tens or hundreds of milliseconds [75,76], and has influences on network parameters, including the efficacies of neurotransmission, synchronicity, and plasticity [77,78,79]. Synaptotagmin-7 [73,79] and Doc2 [80,81] are reported as being high-affinity Ca^2+^ sensors for asynchronous release. Both Doc2 and synaptotagmin-7 exhibit the slow Ca^2+^-regulated membrane-binding kinetics and high affinities required for asynchronous release [80,82,83].

Synaptotagmin-7 mediates asynchronous release in cultured hippocampal neurons [73], and in granule cell synapses as well as in inhibitory synapses formed between basket cells and Purkinje neurons in the cerebellum [84,85], and in excitatory neocortex synapses formed between pyramidal cells and Martinotti neurons [86]. The role of synaptotagmin-7 in asynchronous release is usually only apparent when more than one stimulus is applied [73,85]. At the neuromuscular junction of *Caenorhabditis elegans*, synaptotagmin-3 triggers delayed Ca^2+^-dependent neurotransmitter release following fast synaptotagmin-1-mediated release. The fast and slow properties of neurotransmitter release are due to essentially different C2 domains in synaptotagmin-1 and -3 [87].

Synaptotagmin-7 underlies phasic somatodendritic dopamine release and its Ca^2+^ sensitivity in the substantia nigra pars compacta. In contrast, synaptotagmin-1, underlying axonal dopamine release, plays a role in tonic dopamine release. However, synaptotagmi-1 can facilitate phasic dopamine release after synaptotagmin-7 deletion [88]. These results indicate that synaptotagmin Ca^2+^ sensors subserve different aspects of the transmitter release processes.

Doc2α mediates asynchronous release in cultured hippocampal neurons, both after single AP and during AP trains [80,83,89,90]. Loss of asynchronous release in Doc2α deficient neurons can be rescued by Doc2β [80]. Inconsistently, Purkinje cells to deep cerebellar nuclei synapses [91], nor autaptic cultured hippocampal neurons lacking Doc2 shows no significant changes in asynchronous release [81]. At excitatory synapses in mouse hippocampus, the major Ca^2+^ sensor for asynchronous release is Doc2α, while synaptotagmin-7 supports this process through the activity-dependent docking of SVs [92].

### 3.3. Regulation of the Prefusion Complex

Munc18 binds to free syntaxin-1A, and the heterodimeric complex prevents the ternary SNAREs formation [93,94]. The syntaxin/Munc18 complex is forwarded to the ternary trans-SNAREs catalyzed by Munc13 [54,95,96]. Munc13 promotes the assembly of the SNAREs in cooperation with Munc18, forming the parallel configuration of all components of the SNAREs [50], suggesting that Munc13 and Munc18 are assembly factors for establishing the ternary SNAREs. Both syntaxin-1A (membrane SNARE) and synaptobrevin-2 (vesicle SNARE) weakly interact with the MUN domain of Munc13 [50,55,97,98]. An interaction between synaptobrevin-2 in the membrane proximal region and Munc13 in the MUN domain is essential for the function of Munc13 [55]. The efficiency of Ca^2+^-triggered SV fusion is significantly increased by Munc18 and Munc13 in a reconstituted fusion assay [50].

### 3.4. Disassembly of the Postfusion SNAREs

After SV fusion, the ternary SNARE complex is disassembled for recycling the individual SNARE proteins. The ATPase NSF disassembles the ternary SNARE complex, with ATP hydrolysis cooperating with the adaptor protein, SNAP [99,100,101]. These catalyzing molecules of NSF and SNAPs interacting with ternary SNAREs form the so-called 20S complex, for starting state of the disassembly process. However, the molecular mechanism for NSF-mediated SNARE complex disassembly is not yet clarified [11].

High-resolution reconstruction of the NSF, αSNAP, and the full-length soluble neuronal SNARE complex (composed of syntaxin-1A, synaptobrevin-2, SNAP-25A) demonstrated the molecular interactions between NSF and αSNAPs with the SNAREs as follows. [102] Electrostatic interactions by which two αSNAP molecules interface with a specific surface of the SNARE complex. This interaction positions the SNAREs such that the 15 N-terminal residues of SNAP-25A are loaded into the ring pore of NSF via a spiral pattern of interactions between a conserved tyrosine NSF residue and SNAP-25A backbone atoms. This loading process likely precedes ATP hydrolysis. Subsequent ATP hydrolysis then drives complete disassembly. Details of the molecular mechanisms for SNARE complex disassembly machinery have been reported in a review [11]. 

## 4. Replenishment of Release Site with Synaptic Vesicles

### 4.1. SV Dynamics after AP

The ‘zap-and-freeze’ method, generating an AP and following high-pressure freezing at defined time points, can enable characterization of the spatial and temporal organization of the SV fusion sites following an AP firing [13]. With this technical approach, SV dynamics were morphologically analyzed at milliseconds time points after AP in presynaptic terminals of mouse hippocampal neurons in culture. 2% of the synaptic profiles in unstimulated synapses showed exocytic pits in the AZ. Within 5 msec after AP, the synchronous fusion of multiple SVs occurred throughout a single AZ. During synchronous fusion, docked SVs reduced by ~40%, in contrast, SVs close to the membrane (between 6 and 10 nm) slightly increased. Such SVs are possibly in a “loose state”, with SNAREs, synaptotagmin-1, and Munc13 still being engaged [103]. At 5 msec, 18% of the synaptic profiles showed exocytic pits in the AZ. By 11 msec, fused SVs collapsed into the plasma membrane. From 5 to 11 msec, asynchronous fusion followed in the center of the AZ. During asynchronous fusion, the docked SVs are not further depleted in spite of their ongoing fusion, suggesting an active recruitment of SVs during this process. At 14 msec, the docked SVs are fully restored to prestimulus levels with newly docked SVs. This fast recovery of docked SVs is Ca^2+^-dependent, and temporary lasting for 100 msec or less. During the recovery period, newly docked SVs undock or fuse, indicating that the sequence of rapid redocking and subsequent slow undocking may underlie the synaptic facilitation.

The series of snapshot images taken by the ‘zap-and-freeze’ method demonstrated millisecond SV dynamics, such as synchronous and asynchronous fusion, undocking, and docking, which are regulated by AZ proteins as discussed in Section 2.2, following transient Ca^2+^ elevation, with the opening of Ca_V_ channels accompanying AP.

### 4.2. AZ Proteins

AZ proteins contribute to the establishment of multiple functionally definable stages of the SV state, as discussed in Section 2.2. For the replenishment of release site with SVs, possible functions of RIM-BP, CAST, Bassoon, and Piccolo have been reported.

RIM-BP, interacting with RIM and Ca_V_ channels [58], organizes the SV release site topography [37,60]. RIM-BP in *Drosophila* supports a rate-limiting stage required for the replenishment of high release-probability SVs that follows depletion of SVs [104]. In a mammalian auditory synapse of the cochlear nucleus RIM-BP controls both the release probability and the SV replenishment [31]. Loss of RIM-BP2 lowered the release probability, due to a slowed down of the Ca^2+^-dependent fast SV replenishment. The ultrastructural studies revealed reduced docked SVs and proximal SVs, in addition to an impaired Ca_V_ channels topography in the AZ [31]. Ca_V_2.1 channel localization in the AZ is specifically controlled by RIM-BP binding to Bassoon [105]. Thus, it is likely that RIM-BP, interacting with Bassoon via RIM, controls the rate of Ca^2+^-dependent fast SV replenishment at the fast central auditory synapse [31].

CAST/ELKS also controls SV replenishment [15]. In cultured sympathetic presynaptic terminals, CAST^S45^ is phosphorylated in an activity-dependent manner. Expression of the phosphomimetic-CAST reduced the SV number in the RRP. The paired-AP protocol experiments indicate that the phosphorylation of CAST^S45^ causes paired-EPSP depression with a time window of 30–120 msec after the first AP. Overexpression of the phosphonegative-CAST reduced the paired-EPSP depression (<200 msec), suggesting that phosphorylated CAST^S45^ downregulates SV reloading shortly after AP, but not over a longer period. The possible kinase is a serine/threonine kinase SAD-B, a presynaptic kinase that is associated with the AZ cytomatrix and SVs, and that phosphorylates CAST^S45^ in vitro [15]. Acute deletion of CAST significantly delayed the rate of fast SV reloading, following the RRP depletion with AP bursts. These results indicate that CAST is required for fast SV reloading; however, the phosphorylated CAST, within 200 msec after AP, brakes transmitter release by slowing down SV reloading. The braking of SV reloading may save SVs for an incoming AP to the presynaptic terminal. RIM1, a binding partner of CAST, interacting with Munc13-1, is implicated in SV docking and priming [106]. These protein interactions are likely involved in the CAST-mediated fast replenishment of release sites with release-ready SV.

Bassoon participates in the reloading of SVs to release sites in excitatory synapses of cerebellar mossy fiber connecting to granule cell [40]: Bassoon knockout enhanced short-term synaptic depression during sustained high frequency stimulation, halving the SV reloading rate, whereas it caused no effect on basal synaptic transmission. At the central endbulb synapse in Bassoon knockout mice, SV replenishment rate was slowed down, whereas vesicle number and the accompaniment were normal [19]. These results suggest a role for Bassoon in speeding up high activity-dependent SV tethering, leading to the rapid replenishment of release sites. At the rat calyx of the Held synapse, Bassoon and Piccolo separately or simultaneously share functions in the SV replenishment during high-frequency synaptic activity [41]. In Piccolo-lacking calyxes, the recruitment of slowly releasing SVs in the RRP, that is normally invisible for AP-induced release, is visible during high-frequency stimulation, indicating a role for Piccolo in establishing a sub-pool of the RRP for preventing depletion of release-ready SVs during prolonged and intense firing activity [41]. Additive roles of Piccolo and Bassoon in SV replenishment are revealed in the fast central auditory synapse: Piccolo unlikely influence the release probability, while Bassoon likely regulate it [42].

### 4.3. Motor Proteins

For filling up the RRP during sustained neural signals of AP, motor proteins are proposed to translocate SVs from a larger SV cluster, called the recycling pool or reserve pool [107,108]. However, electrophysiological studies on the calyx of Held synapses [109] and the cerebellar synapses of the parallel fiber and the molecular layer interneuron [110] demonstrated that myosin II controls SV dynamics in the RRP. In these synapses, the RRP consist of two pools, a fast- and a slow-releasing pool [110,111]. In response to AP trains, myosin II translocates SVs with a rapid rate constant from slow-releasing SVs to fast-releasing ones [109,110]. The fast- and slow-releasing SVs pools may correspond to the docked SVs and the undocked SVs observed morphologically after a single AP [13]. These two SVs pools can be defined under the APs train: A single fast component exists at train onset, while both a fast and a slow component exist later in the train [110].

**Figure 2:**
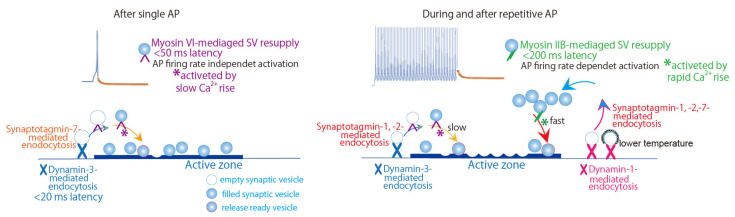
Schematic drawing of a possible linkage of endocytosis to exocytosis through activity-dependent distinct synaptic vesicle recycling pathways in presynaptic sympathetic neurons. (Left) After single action potential (AP), within 20 msec, an endocytic protein, dynamin-3, mediates ultrafast endocytosis, and within 50 msec, a motor protein, myosin VI, resupplies synaptic vesicles (SVs) to the release site. For ultrafast endocytosis, synaptotagmin-7 likely acts as a Ca^2+^ sensor. (Right) During and after repetitive APs, recovery pathways of release-ready vesicles with distinct kinetics involves dynamin isoforms, Ca^2+^ sensors, and myosin isoforms. For fast recovery, dynamin-1 and myosin IIB are involved, and synaptotagmin-1, -2, and -7 can be mediators for endocytosis. In contrast, for the slow recovery pathway, dynamin-3 and myosin VI are involved, and synaptotagmin-1 and -2 can be mediators for endocytosis. Adapted from Lu et al., 2009 [116], Tanifuji et al., 2013 [114], Mori et al., 2014 [117], Hayashida et al., 2015 [113], and Tanifuji’s unpublished data.

We have demonstrated roles for myosin isoforms in transmitter release from presynaptic terminals of sympathetic neurons, where myosin IIB and myosin VI are specifically expressed [112,113]. Myosin IIB is activated by myosin light chain kinase via Ca^2+^/calmodulin (CaM), while VI is directly activated by Ca^2+^/CaM. Myosin IIB or VI participation in replenishment of the RRP with SVs can be predicted from the recovery kinetics of release-ready SVs under various firing patterns [113]. Application of the paired-AP protocol shows synaptic depression at short intervals of APs within 120 msec. Myosin IIB deletion shows no change in the paired-EPSP size, while myosin VI deletion potentiates the decrease in the paired EPSP at ≥50 msec intervals. These observations indicate that an AP within 50 msec activates myosin VI (Figure 2), but not myosin IIB, and that, within 120 msec of the AP, myosin VI completes refilling of the release site with release-ready SVs. Application of AP train at 10 Hz, revealed that myosin IIB takes 200 msec or needs two APs for the SV replenishment, while myosin VI reloads SVs within 100 msec of AP. More frequent APs seem to activate myosin IIB. 

Our studies indicate that, during and after intense firing in sympathetic neurons, the SV replenishment of release sites is achieved through distinct pathways (Figure 2). The SV reloading kinetics displays fast and slow phases, due to different molecular contributions [113,114,115,116,117]. Myosin IIB deletion moderates the fast recovery, but not the slow recovery, whereas VI deletion accelerates the fast recovery and decelerates the slow recovery [113]. Both deletion substantially delay both the fast and slow recoveries [113]. Thus, myosin IIB and VI mediate the SV replenishment through the fast and slow SV resupply pathways, respectively, during and after repetitive APs.

## 5. Presynaptic Plasticity

### 5.1. Presynaptic Short-Term Plasticity

During and after repetitive presynaptic AP activity presynaptic short-term synaptic plasticity occurs on a timescale of milliseconds to minutes, and it modulates synaptic efficacy changing the release probability and the RRP size [118].

RIM1 participates in presynaptic short- and long-term synaptic plasticity [119,120]. At cerebellar parallel-fiber synapses, RIM1α deletion decreases the release probability, in consequence enhances short-term facilitation, whereas the long-term plasticity is fully intact [120]. In hippocampal neurons, RIM1α function depends on different synapses: At the CA1 region Schaffer-collateral excitatory synapses and in GABAergic synapses, RIM1α is required for short-term plasticity, while, in excitatory CA3-region mossy fiber synapses and cerebellar parallel fiber synapses, RIM1α is required for presynaptic long-term plasticity. This long-term plasticity depends on the phosphorylation of RIM1α, suggesting that RIM1α acts as a ‘phosphoswitch’ for setting the synaptic strength [119]. In addition to RIM1, RIM-BP may participate in short-term plasticity by controlling the Ca^2+^-dependent fast SV replenishment [31].

As described above the release probability is controlled by RIM, while enlargement of the SV pool size depends on Munc13 action [106]. The participation of Munc13 to presynaptic short-term plasticity has been proposed [14,103]: Calmodulin binding to Munc13 proteins increases its priming activity and RRP sizes in autaptic synapses of hippocampal neurons. The Ca^2+^ sensor/effector complex, that is activated by residual Ca^2+^ elevation during and after repetitive AP, shapes short-term plasticity [14]. In the calyx of the Held synapses, the Ca^2+^ sensor/effector complex also determinate the characteristics of short-term synaptic plasticity [103]. Furthermore, calmodulin-activated Munc18-1 contributes to presynaptic short-term plasticity, such as paired-pulse facilitation, in sympathetic neurons [121]. In addition to Munc13 and Munc18, other AZ proteins are possibly targets of Ca^2+^–calmodulin during and after repetitive AP, and could promote facilitation of transmitter release. Activation of Munc13 by phorbol esters is also reported an essential reaction for synaptic potentiation [122]. Phorbol esters are analogues of diacylglycerol that activates PKC, however, Munc13-mediated synaptic potentiation in hippocampal autaptic neurons is PKC-independent [123]. Instead, Munc18-1 mediates the synaptic potentiation as a downstream target in the PKC pathway [123].

Bassoon and Piccolo modulate short-term depression during high AP activity in the rat calyx of the Held synapse and in the cerebellar mossy fiber to granule cell synapse [40,41]. In the calyx of the Held synapse Piccolo contributes to the slow-releasing SVs replenishment that is unengaged in AP-induced release during high-frequency stimulation. Therefore, control of the SV reloading into the RRP during sustained synaptic activity in the calyx of the Held synapse is shared by functions of Bassoon and Piccolo, whereas Bassoon seems to be more efficient [41].

During sustained high-frequency synaptic activity, RIM1, Munc13, Munc18, Bassoon, and Piccolo participate in the short-term synaptic plasticity. In contrast, CAST and CAST^S45^ phosphorylation by SAD-kinase contribute to milliseconds short-term depression after a single AP in cultured sympathetic neuron synapses. CAST^S45^ phosphorylation potentiates the paired-pulse depression (see Section 4.2) [15].

### 5.2. Presynaptic Long-Term Plasticity

Post-tetanic potentiation (PTP) has been traditionally described as being a form of short-term plasticity that decays within tens to hundreds of seconds [118]. However, recent reports of PTP studied in the hippocampal mossy fiber synapses termed it as being presynaptic long-term plasticity. Recent morphological studies with the “flash and freeze” analysis demonstrated rapid rearrangement of ultrastructure at potentiated synaptic boutons: SVs spread in the bouton and accumulated in the AZ, and the AZ density and synaptic complexity increased as well. Thus, the induction of PTP, which increases synaptic strength, relies on the rapid ultrastructural remodeling [124]. An increase in docked SVs and that in the size of the RRP has been proposed as a mechanism for PTP [125] that is dependent on the presynaptic increase of cAMP [124,126]. Two-dimensional time-gated stimulated emission depletion microscopy showed an increase in the P/Q-type Ca^2+^ channel cluster size near the release sites, suggesting a potential mechanism for the cAMP-dependent increase in transmission at hippocampal mossy fiber synapses, namely an accumulation of the Ca^2+^ channels in the AZ [127]. RIM1, a priming factor as described in Section 2.2, is crucial for cAMP-dependent long-term potentiation [128]. Furthermore, the trans-synaptic modulation of presynaptic shot-term plasticity in hippocampal mossy fiber has recently reported [129].

### 5.3. Presynaptic Homeostasis Plasticity

Bruchpilot, a *Drosophila* orthologue of CAST/ELKS and essential for Ca^2+^ channel integrity and AZ structural conservation [130,131], has been proposed to participate in homeostatic presynaptic potentiation. Bruchpilot forms normally a ring comprising a T-bar per AZ; however, it forms multiple rings in the AZ of the glutamate receptor-deficient synapse [17]. In response to changes of synaptic activity synaptic connections undertake homeostatic readjustment to secure a stable and flexible nervous system. Homeostatic signaling has demonstrated in both the central and peripheral nervous systems in various species from *Drosophila* to humans [17,129,132,133,134]. At the endplates of myasthenia gravis patients, for example, to keep muscle excitation reduced postsynaptic sensitivity is balanced out by upregulated neurotransmitter release [135]. Thus, AZ proteins could functionally participate in the homeostatic regulation system by regulating the SV localization and the SV states.

Among AZ proteins, RIM [18,136], RIM-BP [104], and Bassoon [19] have been reported to contribute to homeostasis plasticity. During homeostatic synaptic plasticity at the *Drosophila* neuromuscular junction, RIM participates in the RRP enlargement [18]. RIM-BP is essential for the homeostatic neurotransmitter release modulation as well, and it participates in both the Ca^2+^ influx enhancement and the RRP enlargement [104]. RIM-BP is required for the normal refilling of high release-probability SVs, which is independent of the Ca^2+^ influx and the RRP size modulation [104]. Thus, a presynaptic vesicle pool of high release-probability SVs locating near Ca^2+^ channels and RIM-BP in the AZ might be a target of the presynaptic homeostatic mdulation. The high release-probability SV might comparable to the primed SV in mammalian synapses (see Section 2.2). In the fast central endbulb synapse of auditory nerve fibers to bushy cells of the cochlear nucleus, the Bassoon dysfunction slows down the SV replenishment and induces homeostatic plasticity [19]. There, although SV replenishment and RRP are reduced, quantal size, vesicular release probability, and postsynaptic densities are increased, suggesting that presynaptic dysfunction drives homeostatic plasticity both in presynaptic and postsynaptic functions for synaptic upscaling.

## 6. Synaptic Vesicle Endocytosis

SVs are recycled within nerve terminals. After exocytosis, SVs are recovered by either fusion pore closure at the AZ [137,138], later termed as “kiss-and-run” [139], or clathrin-mediated endocytosis directly from the plasma membrane at the periphery of the AZ [140]. In addition to these classical observations, ultrafast endocytosis at the edge of the AZ provides SVs from the synaptic endosome at low neural activity [141,142]. During high neuronal activity, bulk endocytosis retrieves the internalized plasma membrane [143], and SVs are regenerated from the bulk endosome [144]. It is possible that all four mechanisms co-exist in nerve terminals and are used differently, depending on the activity levels or the synapse types [145].

### 6.1. Kiss-and-Run

Using electron microscopy, Ceccarelli et al. reported that clear vesicles were potentially internalized at the AZ in the electrically stimulated frog neuromuscular junction [137,138], and proposed that SVs can be recycled by the reversal of an exocytic fusion pore, a model that was later termed “kiss-and-run” [139]. Kiss-and-run is a mode of the SV fusion and rapid retrieval without the full collapse of the SV [146,147]. The high-speed imaging of quantum dots was employed to explore the single-SV collapse and reuptake [148], and supported this mode of endocytosis. Loading individual SVs with single quantum dots into hippocampal neurons, Tsien and coworkers demonstrated that kiss-and-run dominated at the beginning of stimulus trains, reflecting the preference of vesicles with a high release probability. Its incidence was increased by rapid firing, a response that was appropriate for shaping the kinetics of neurotransmission during a wide range of firing patterns [148]. Other optical approaches also demonstrated kiss-and-run endocytosis at mammalian central synapses [5,149] which maintains its molecular identity bypassing the need for the endocytic sorting of SV proteins or their passage through endosomal intermediates [150].

### 6.2. Clathrin-Mediated Endocytosis

Using electron microscopy, Heuser and Reese reported that SVs are regenerated locally by the formation of clathrin-coated vesicles at the periphery of the AZ in the electrically stimulated frog neuromuscular junction [140]. This mode of endocytosis has been extensively studied in invertebrates such as nematodes, fruit fly, and squid; and in vertebrates such as lampreys and rodents [5]. In contrast to kiss-and-run, clathrin-mediated endocytosis is retrieval of the full collapsed SVs into the plasma membrane for the regeneration of SVs. Clathrin-mediated endocytosis is relatively slow (10–30 s), in contrast to the fast kiss-and-run (<1–2 s) [5], and requires a set of proteins, clathrin and clathrin-associated proteins [151,152]. Details of the molecular mechanisms for clathrin-mediated endocytosis have been reported in reviews [151,152,153].

Recently, it is suggested that, at a physiological temperature in mammalian neurons, endocytosis occurs without formation of clathrin-coated vesicles [144,154,155]. Endocytosis proceeds within the neuron knocked-down clathrin heavy chain or its adaptor, AP-2 [144,156,157]. The clathrin-coated vesicles are proposed to regenerate SVs from endosomes [6,144].

### 6.3. Ultrafast Endocytosis

Ultrafast endocytosis completes in as fast as 50 msec after evoked exocytosis, and continues stochastically for seconds [141,142,154]. This mode of endocytosis, demonstrated in the *Caenorhabditis elegance* neuromuscular junction [142] and mouse hippocampal neurons [141,154], is predominant at a physiological temperature. The membrane at the lateral edges of the AZ invaginates and forms a large endocytic vesicle (approximately 80 nm) which is immediately delivered to the synaptic endosomes. From the synaptic endosome, SVs are regenerated by budding in a clathrin-coated form. Ultrafast endocytosis seems to rely on the proper maintenance of membrane tension [158], and on the immediate clearance of the AZ membrane [159,160].

Ultrafast endocytosis shares many molecular players with other endocytosis processes, including the clathrin-mediated process. Forming a narrow neck on the budding vesicle from the membrane, synaptojanin-1 [161] and endophilin-A [162] coordinately tubulate the invaginated membrane [145]. Its pinch-off is mediated by dynamin-1 and actin [141,142]. This endocytosis is strongly influenced by membrane fluidity, which is under the control of temperature [144].

### 6.4. Bulk Endocytosis

Single-SV retrieval modes, such as clathrin-mediated endocytosis, predominate under mild synaptic activity. In contrast, under intense activity, additional SV retrieval mode, termed the activity-dependent bulk endocytosis, is activated [163]. It is the dominant and a high-capacity SV retrieval mode under higher synaptic activity, and plays key roles in neurotransmission [157]. [163]. This mode of endocytosis is observed at invertebrate, amphibian, and mammalian synapses [143,145,164,165], and in vivo, at a central synapse in awake rats [166].

A large area of invaginated membrane is retrieved within 1–2 s, and forms a bulk endosome (average 150 nm) [153,163], from which functional SVs are generated [167]. This membrane retrieval is clathrin-independent, but dependent on calcineurin, a Ca^2+^-dependent protein phosphatase [153]. Actin is required for the large area of membrane invagination [155,168]. SV proteins such as VAMP2, synaptophysin, and vesicular glutamate transporter are retrieved by this mode of endocytosis [157,169]; in contrast, some SV proteins such as VAMP4 are accumulated by activity-dependent bulk endocytosis [169]. Noncanonical v-SNAREs such as VAMP4 [170] or VAMP7 [171,172] drive spontaneous SV fusion, suggesting a possible retrieval system for each SV protein.

### 6.5. Ca^2+^-Sensors Link Exocytosis to Endocytosis

The four modes of endocytosis summarized above are neuronal activity-dependent. Ultrafast endocytosis can be triggered by a single AP [13]. Although the other modes of endocytosis were triggered by trains of APs, kiss-and-run dominated at the beginning of the stimulus trains [148]. These observations suggest low-affinity Ca^2+^ sensors likely link exocytosis to endocytosis. Synaptotagmin-1 dysfunction impairs SV endocytosis in the *Drosophila* neuromuscular junction [173]. In rat hippocampal neurons synaptotagmin-1 is also required for single- as well as multi-vesicle endocytic events [174]. Synaptotagmin-7, a Ca^2+^ sensor for asynchronous release [73,175,176], is in charge of the slowed endocytosis seen under synaptotagmin-1 dysfunction [174].

Dynamin, a key protein in most modes of endocytosis [177], mediates the fission of internalized plasma membrane, and vesicle scission from synaptic endosomes [5]. A possible linkage of Ca^2+^ sensors to vesicle endocytosis was examined in presynaptic sympathetic neurons. There, dynamin-3 is responsible for the ultrafast endocytosis that is activated within 20 msec of a single AP (Figure 2) [114]. For this ultrafast endocytosis, synaptotagmin-7 likely act as a Ca^2+^ sensor (Tanifuji and Mochida, unpublished data). The synaptotagmin-7 deletion potentiated the paired-pulse depression to a similar degree of the potentiation caused by dynamin-3 deletion. Both synaptotagmin-7 and dynamin-3 deletion remarkably induced the failure of paired EPSP, supporting a possible role for synaptotagmin-7 in triggering ultrafast endocytosis. 

During and after repetitive APs, dynamin-1 mediates the fast SV recycling, while dynamin-3 mediates the slow SV recycling (Figure 2) [114]. The fast SV recycling was similarly delayed with synaptotagmin-1, -2, or -7 deletion, while the slow SV recycling was delayed with synaptotagmin-1 or -2 deletion. Taken together, both synaptotagmin-1 and -2 are related to endocytosis under physiological frequency neuronal activity, while synaptotagmin-7 is mainly required for the ultrafast endocytosis after a single shot of AP. These distinct mediations of the SV recycling (Figure 2) might be due to specialized localizations of Ca^2+^ sensors: Synaptotagmin-1 and -2 are expressed on the SV membrane, while synaptotagmin-7 is more concentrated on the presynaptic plasma membrane or internal membrane [178,179]. 

## 7. Conclusions

The AZ protein assembly promotes SV states of tethering, docking and priming, and it sets release-ready SVs nearby Ca^2+^ channels in the RRP [1]. The release-ready primed SV proceeds to SV fusion by AP. Exocytosis is a series of events controlled by protein–protein interactions: Fusogenic SNAREs, the Ca^2+^-sensor synaptotagmin, the activator/regulator complexin, the assembly factors Munc18 and Munc13, and the disassembly factors NSF and SNAP are the core molecules of the fusion machinery [11]. AP-evoked sub-millisecond SV fusion occurs releasing the complexin-mediated inhibition by Ca^2+^-bound synaptotagmin [38,65]. Synaptotagmin-1 mediates fast and synchronous SV fusion [38,65]. Synaptotagmin-2, at the calyx of the Held synapse and in some GABAergic neurons, also mediate fast and synchronous SV fusion redundantly with synaptotagmin-1 [71,72]. Synaptotagmin-3 [87] and -7 [73,79], and Doc2 [80,81] are high-affinity Ca^2+^ sensors for slow and asynchronous SV fusion.

AP triggers SV dynamics in the AZ, following millisecond Ca^2+^ dynamics that control SV states, synchronous and asynchronous fusion, and undocking, redocking, and priming [13]. The undocking, redocking, and priming of SV states contribute to presynaptic short-term plasticity [13]. AP-induced millisecond Ca^2+^ dynamics activate multiple protein cascades via Ca^2+^-sensor molecules [180] to control the replenishment of the release site with release-ready SVs [7]. Neurotransmitter release probability, controlling SV/Ca^2+^ channel coupling, is regulated by RIM and RIM-BP, which are required for clustering the Ca^2+^ channel at the release site [29,30,31]. The RRP size, which controls the SV states of docking and priming, is regulated by CAST/ELKS and Munc13, respectively [15,53,106]. During and after repetitive AP firing, Bassoon and Piccolo play a role in SV tethering under sustained high AP activity, to maintain sustainable synaptic transmission [19,40,42].

Presynaptic plasticity is dependent on flexible neurotransmitter release. Residual Ca^2+^-dependent regulation of the release probability via SV fusion machinery has been proposed for the generation of presynaptic plasticity [118]. However, as reviewed in this article, the contribution of AZ proteins to presynaptic plasticity is significant [119,120,121,122,123,124,125,126,127,128]. Synaptic connections undergo homeostatic readjustment in response to changes in synaptic activity, to ensure a stable and flexible nervous system [17,129,132,133,134]. Important roles of RIM [18], RIM-BP [104], and Bassoon [19] in presynaptic homeostatic plasticity, which control the release probability and the RRP, have been proposed so far. Stable and flexible homeostatic synaptic transmission for non-stop signaling is likely supported by the coordinated functions of RIM, RIM-BP, Bassoon, and other AZ proteins.

SVs are recycled within nerve terminals. After exocytosis, the SVs are recovered via either fusion pore closure “kiss-and-run” [139] at the AZ [137,138], or via clathrin-mediated endocytosis directly from the plasma membrane at the periphery of the AZ [140]. In addition to these classical observations, ultrafast endocytosis at the edge of the AZ provides SVs from the synaptic endosome at a low level of neural activity [141,142]. Ultrafast endocytosis is highly temperature sensitive. During high neuronal activity, bulk endocytosis retrieves the internalized plasma membrane [143], and the SVs are regenerated from the bulk endosome [144]. Under lower levels of activity and significantly lower than physiological temperature, the most endocytic membrane retrieval is clathrin-mediated. At a physiological temperature clathrin-mediated budding may be relocated to rapidly formed endosomes [144]. All four endocytic mechanisms possibly co-exist in presynaptic terminals, and are activated under different conditions of neuronal activity and temperature [145].

For summarizing this review discussing the mechanisms of SV exo- and endocytosis, molecular players act in the presynaptic release site AZ, the exocytosis, the replenishment of the release site with SVs, and the endocytosis are listed in the Table 1. 

## Figures and Tables

**Table 1:** Molecular players act in the presynaptic release site active zone, synaptic vesicle exocytosis, replenishment, and endocytosis.

Function			Protein	References
AZ protein complex	AZ assembly	Ca_V_ channel recruitment	RIM, RIM-BP, CAST/ELKS	[30,31,33]
		liquid droplet formation	RIM, RIM-BP, ELKS	[37,59]
		stabilization and degradation	Bassoon, Piccolo	[64]
	SV states	tethering	Bassoon, Piccolo	[40,41,42]
		docking	RIM, CAST/ELKS	[3,15]
		priming	ELKS, RIM, RIM-BP, Munc13	[48,49,50,51]
		super-priming	Mover	[52]
		fusion	Munc13, Munc18	[53,54,55,56,57]
	Fusion machinery interaction	fusion machinery regulation	Munc13, Munc18	[53,54,55,56,57]
Synaptic vesicle exocytosis	SV fusion complex	fusion machinery	SNAREs	[1,2,11,39]
		Ca^2+^ sensor	Synaptotagmin-1 Synaptotagmin-2 Synaptotagmin-7	[1,2,11,38,39,65] [71,72] [73]
		regulator	Complexin-1	[65,66,67,70]
		assembly factor	Munc13, Munc18	[50,53,54,55,56,57,93,94,95,96]
		disassembly factor	NSF, SNAP	[99,100,101,102]
	Asynchronous SV fusion	Ca^2+^ sensor	Synaptotagmin-7 Synaptotagmin-3 Doc2α	[73,84,85,86] [87] [80,83,89,90,92]
Synaptic vesicle replenishment	AZ proteins	facilitation	RIM-BP, Bassoon, Piccolo,	[31,40,41,104]
		inhibition	CAST phosphorylation	[15]
	Motor proteins	facilitation	Myosin II	[109,110,113]
		facilitation	Myosin VI	[113]
Presynaptic plasticity	Short-term plasticity	short-term plasticity	RIM1α, Munc13	[14,103,106,119,120,122]
		post-tetanic potentiation	Munc18	[123]
		control of depression	Bassoon, Piccolo	[40,41]
		depression	CAST phosphorylation	[15]
	Long-term plasticity	cAMP-dependent increase in transmission	RIM1, P/Q-type Ca^2+^ channel	[119,120,124,126,127,128]
	homeostatic plasticity	RRP enlargement	RIM	[18,136]
		promotion of SV priming	RIM-BP	[104]
		promotion of SV replenishment	Bassoon	[19]
Synaptic vesicle endocytosis	kiss-and-run			[5,139,146,147,148,149]
	clathrin-mediated		Clathrin-associated proteins	[5,140,151,152,153]
	ultrafast endocytosis		Synaptojanin-1, endophilin-A Dynamin, Actin	[141,142,145]
	bulk endocytosis		Calcineurin, Actin	[153,155,168]
	Ca^2+^ sensors		Synaptotagmin-1 Synaptotagmin-7	[173,174] [174]

## Data Availability

Not applicable.

## Abbreviations

AP	action potential
AZ	active zone
CAST	cytomatrix at the active zone-associated structural protein
CaM	calmodulin
Ca_V_ channels	voltage-gated Ca^2+^ channels
EPSPs	excitatory postsynaptic potentials
PKC	protein kinase C
PTP	post-tetanic potentiation
RIM	Rab3-interacting molecules
RIM-BP	RIM-binding protein
RRP	readily releasable pool
SNAREs	soluble *N*-ethylmaleimide-sensitive-factor attachment receptor proteins
SUVs	small unilamellar vesicles
SVs	synaptic vesicles
